# Challenge in RBORL History

**DOI:** 10.1016/S1808-8694(15)31234-9

**Published:** 2015-10-20

**Authors:** Silvio Caldas Neto

**Affiliations:** Publication Director ABORL-CCF

It is a delicate task to write the first editorial of the Brazilian Journal of Otorhinolaryngology. Owing to its rich scientific content and feature of interesting articles, RBORL has a fascinating history. It is this history that challenges us. The Sao Paulo Journal of Otorhinolaryngology was founded in January 1933. At the time, its Editors were focusing on putting aside “bitter criticism” that was given to them by publishing the article “regionalism act”. In the first years of the 1950's, the Journal was incorporated into the Brazilian Federation of the Societies of Otorhinolaryngology and Bronchoesophagology. As expected, it became a national journal, owing to its new and definitive denomination. It is curious (and maybe not a mere coincidence) that the journal we originally obtained our name from was founded in Recife in 1935, as the Journal of Anais Otorhinolaryngology - a scientific periodical of national circulation highly acclaimed by otorhinolaryngologists in Recife and in the Northeast of Brazil.

Since then, the Journal has made an upward curve and more recently – why not? – a sharp curve. It is not surprising that this publication has been coordinated by people such as: Mário Ottoni de Rezende, Homero Cordeiro, José Rezende Barbosa, Rudolph Lang, Arthur Kós, Fernando Portinho, Ricardo Bento, Paulo Pontes, Carlos Alberto Campos and Henrique Olival Costa (not to mention those who have actively assisted them, such as Nicanor Letti, Priscila Bogar, among others).

The Brazilian Journal of Otorhinolaryngology has adapted to global changes following various parameters regarding scientific publication. One of these changes is that periodicals have been steadily edited in a highly professional way. In addition, it has been easy to understand the journal scientific articles, which point out the level of scientific knowledge of researchers, graduate studies programs and universities. Furthermore, the considerable progress of RBORL not only reflects the ascension of Otorhinolaryngology as a medical specialty in Brazil, but also demonstrates great intellectual development in the country.

We need to highlight that we are undertaking the challenging task of coordinating ABORL-CCF Board of Publications when our Journal finds itself in the final stage of indexation by Medline. It is an outstanding merit to all individuals who have managed it so far, more specifically to Henrique Olival, who hands over the Journal among the top journals in the Brazilian Medical Publishing Houses. In addition, he provides us with an extremely workable and effective editorial management system, which has been recently implemented and might need few improvements to meet the long run needs of contributors.

It is necessary to index a journal in a database if we want to pursue visibility and prestige, as well as achieve national and international market penetration. Amongst all main databases for Brazilian publications, in order of importance, are: LILACS, SciELO, Medline and ISI (Institute for Scientific Information) that publish journals of major impact. Indexing in Medline and publishing editions in English are part of the last managements' accomplishments. These were decisive steps towards achieving our last goal, which is to increase our impact of 0.0512 points in the past three years, whereas a similar journal, called Acta Cirúrgica Brasileira, has 0.1401 points. Memórias do Instituto Oswaldo Cruz, indexed in ISI, has an impact of 0.2022.

The qualification of a journal and its impact depend on its regularity, periodicity and scientific quality. This last criterion is directly related to original articles and case studies. In the case of RBORL, the above-mentioned criteria are very good, but they can be better. As the number of case studies submitted is great, they generate a major and restrained demand, which we have to find a solution for, without affecting the scientific quality. We find ourselves obliged to examine the relevance of facts and sometimes stop receiving these types of articles. We want to employ stricter methods to review and select articles, which will optimize the publishing time of the approved ones. We are going to provide space for those who wish to publish their case studies in our website. Case studies that have already been submitted will be treated as usual.

The improvement of our indicators is essential to achieve the highest categories of periodical classification of CAPES. This will help us to add more value to our articles, offer more places in graduate studies courses, raise funds, have more decisive influence in universities and university hospitals, and gain more respect to our specialty. This is the path. This is the main challenge presented to us. It is not less than what was faced by Sylvio Caldas (the real one, with a “y”) who, in Recife, driven by the same spirit that has motivated everyone from the RBORL History 70 years ago, founded, managed and gave financial support to Journal of Anais Otorhinolaryngology, using his own resources for three years. This editorial is dedicated to him and to this type of spirit.

